# microRNAs in the Lymphatic Endothelium: Master Regulators of Lineage Plasticity and Inflammation

**DOI:** 10.3389/fimmu.2017.00104

**Published:** 2017-02-09

**Authors:** Daniel Yee, Mark C. Coles, Dimitris Lagos

**Affiliations:** ^1^Centre for Immunology and Infection, Department of Biology, Hull York Medical School, University of York, York, UK

**Keywords:** lymphatic endothelial cells, microRNA, inflammation, lineage plasticity, lymphangiogenesis

## Abstract

microRNAs (miRNAs) are highly conserved, small non-coding RNAs that regulate gene expression at the posttranscriptional level. They have crucial roles in organismal development, homeostasis, and cellular responses to pathological stress. The lymphatic system is a large vascular network that actively regulates the immune response through antigen trafficking, cytokine secretion, and inducing peripheral tolerance. Here, we review the role of miRNAs in the lymphatic endothelium with a particular focus on their role in lymphatic endothelial cell (LEC) plasticity, inflammation, and regulatory function. We highlight the lineage plasticity of LECs during inflammation and the importance of understanding the regulatory role of miRNAs in these processes. We propose that targeting miRNA expression in lymphatic endothelium can be a novel strategy in treating human pathologies associated with lymphatic dysfunction.

## Introduction

The lymphatic system is a transport network that regulates tissue fluid homeostasis, the absorption of macromolecules, and the trafficking of immune cells (1). Lymphatic vessels are made up of a single layer of partly overlapping lymphatic endothelial cells (LECs). Embryonic studies on development of lymphatic vasculature have identified key transcription factors required for development and maintenance of the lymphatic system. The same transcription factors regulate lymphangiogenesis, the process of new lymphatic vessel growth from pre-existing vessels, which has crucial roles in wound healing, inflammation, infection, and cancer. In addition to transcriptional regulation, posttranscriptional mechanisms play a key role in LEC responses to inflammation. In particular, several microRNAs (miRNAs) have emerged as key determinants of LEC differentiation and inflammatory responses. This review will discuss our current understanding of the role of individual miRNAs and components of the miRNA biogenesis machinery in LEC immune function.

## miRNA-Mediated Silencing

microRNAs are a class of highly conserved, small non-coding RNA (~20–24 nt) that regulate gene expression at the posttranscriptional level of all biological pathways including cell development, differentiation, and function (2). In mammals, the canonical process of miRNA biogenesis encompasses the generation of primary miRNA (pri-miRNA) transcripts that are transcribed by RNA polymerase II in the nucleus. Stem-loop structures of pri-miRNA transcripts are processed by the RNAse III endonuclease, Drosha, to form hairpin-shaped precursor miRNA (pre-miRNA) (3, 4). Following this, pre-miRNA is exported into the cytoplasm where it is further processed by another RNAse III endonuclease, Dicer, which cleaves off the hairpin structure. The resultant double-stranded miRNA is separated into two strands with the mature miRNA strand packaged onto the miRNA-induced silencing complex that includes an Argonaute (AGO) effector protein. The miRNA guides RISC to specific target sites, primarily the 3′ untranslated region (UTR) of target mRNAs, leading to repression of target gene expression (5). Binding sites are generally 8mers or canonical sites that enable high miRNA regulation of mRNA expression (6). Due to this short target sequence, miRNAs can have multiple targets, and it is predicted that 30% of all protein-coding genes is under miRNA regulation in mammals (7).

## Embryonic Development and Specification of the Lymphatic Vasculature

Sabin hypothesized the venous origin of the lymphatic system (8), which became increasingly supported by developmental studies around the beginning of the twenty-first century (9). Specific genes for lymphatic differentiation and identity were identified, and these included vascular endothelial growth factor receptor-3 (VEGFR-3), lymphatic vessel hyaluronan receptor-1 (LYVE-1), podoplanin, and prospero-related homeodomain protein 1 (PROX1) (10). VEGFR-3 is a receptor tyrosine kinase for lymphatic-specific VEGF-C and VEGF-D (11). LYVE-1 is a widely used lymphatic-specific marker, implicated in cellular trafficking and a homolog of the CD44 glycoprotein (12, 13). Both VEGFR-3 and LYVE-1 are expressed during early endothelial cell development and become restricted to LECs at later stages. Genetic deletion of VEGFR-3 or VEGF-C in mice leads to defective lymphatic vascular development (14, 15). In contrast, LYVE-1 gene-deficient mice develop normal lymphatic vasculature (16).

The murine lymphatic system begins to form in a subpopulation of venous endothelial cells, LEC precursors, at embryonic day (E) 8.5 that express PROX1, LYVE-1, and VEGFR-3 (14). At E9.75, a lymphatic bias signal upregulates PROX1, LEC budding, and formation of primary lymph sacs (10). PROX1-deficient embryos lack lymphatic vasculature, VEGFR-3, or LYVE-1 expression and are embryonic lethal at E14.5 (10). Two upstream transcriptional regulators of PROX1, SOX18 (17), and COUP-TFII promote the lymphatic bias signal until E13.5 (18, 19). PROX1 and VEGFR-3 continue to be expressed only in postnatal and adult lymphatic vasculature (20). Constant levels of PROX1 are required to maintain LEC lineage, which is supported by VEGF-C/VEGFR-3 signaling (21). Postnatal LECs have lower PROX1 expression compared with embryonic lymphatic endothelium, suggesting low expression of PROX1 is sufficient to maintain LEC identity (22). Additional transcription factors and regulators of lymphatic development have been reported, including neuropilin 2 (23, 24), FOXC2 (25, 26), integrin-9α (27, 28), NOTCH (29, 30), C-MAF (31), and GATA2 (32).

## miRNAs and Endothelial Cell Development

microRNA biogenesis is essential for vertebrate development, and tissue-specificity of miRNAs has been demonstrated in angiogenesis (33–36). Loss of Dicer in mice leads to poor vascular formation and embryonic lethality (33). The highest expressed miRNA in endothelial cells, miR-126 mediates angiogenesis and maintenance of vascular integrity (37–40). Deletion of miR-126 results in vascular leakage, hemorrhaging, and embryonic lethality in a subset of mice (38). Surviving mice lived to adulthood without noticeable abnormalities, suggesting additional regulatory factors after birth. Accordingly, miR-126 targets sprout-related protein-1 (SPRED-1), phosphoinositol-3 kinase regulatory subunit 2 (PIK3R2 also known as P85β), and VCAM-1 in human and murine cells (37–39). By targeting VCAM-1, miR-126 can inhibit leukocyte adherence and potentially regulate vascular inflammation (37). SPRED-1 is an intracellular inhibitor of angiogenic and MAP kinase signaling, and its repression by miR-126 correlated with the increase of pro-angiogenic genes VEGF and fibroblast growth factor in mice (38). Additionally, VEGF can induce miR-132 and promote angiogenesis by suppressing p120RasGAP in human vascular endothelial cells (41).

## Regulation of the miRNA Biogenesis Machinery in LECs

In addition to individual miRNAs, the miRNA biogenesis machinery is regulated during activation of LECs. AGO2 levels are controlled by miR-132 in human LECs (42). Inhibition of miR-132 in activated LECs results in increased AGO2 and the anti-angiogenic miR-221, providing further support for the function of miR-132 in endothelium. Furthermore, activation of TIE-2 by angiopoietin-1 (ANG-1) results in phosphorylation of TRBP (43), a DICER co-factor, which facilitates miRNA processing (44). Through this mechanism, ANG-1 treatment increases levels of miRNAs, including miR-126 and miR-21, which could contribute to the antiapoptotic function of ANG-1 (45, 46) in LECs.

## LEC Plasticity

Altering the levels of PROX1 expression during embryonic, postnatal, or adult stages can reprogram LEC phenotype into blood endothelial cell (BEC) (28, 47, 48). PROX1 deletion results in the upregulation of BEC-specific markers in human and murine LECs (47). Conversely, BECs can be transcriptionally reprogramed by overexpression of PROX1 *in vitro*, resulting in upregulation of VEGFR-3 and podoplanin and suppression of BEC-specific transcripts, such as the transcription factor STAT6 (48, 49). These studies represent that endothelial cell differentiation is reversible and highlight the plasticity of LECs.

## miRNAs and LEC Lineage Commitment

The 3′-UTR of PROX1 is remarkably long (5.4 kb) and conserved among vertebrates (50), which suggests PROX1 expression may be posttranscriptionally regulated by miRNAs. In contrast, the 3′ UTR length of *SOX18* (585 bp) is short and likely to have less miRNA regulation. Profiling of miRNAs in human LECs and BECs led to the discovery that lymphatic development can be regulated by BEC miRNA signatures (40). Overexpression of miR-31 was shown to repress FOXC2 and several other LEC-signature genes (40). Both miR-31 and miR-181a can target PROX1 and as a result repress LEC-specific genes, including VEGFR-3, and vascular development in embryonic LECs (22, 40). Furthermore, signaling from bone morphogenetic protein (BMP) 2, a member of the TGF-β family, inhibited Prox-1 expression and lymphatic differentiation during zebrafish and murine development (51). Interestingly, BMP2 signaling upregulated miRNAs: miR-194, miR-186, miR-99a, miR-92a and also miR-31, and miR-181a (51). Knockdown of SMAD4 by siRNA downregulated the expression of miR-31 and miR-181a indicating a possible involvement of BMP2 as a negative regulator of LEC identity (51). Recently, miR-466 was shown to suppress PROX1 expression and tube formation in human dermal LECs, and both miR-466 and miR-181a induced inhibition of corneal lymphangiogenesis in rats (52).

## LECs in Inflammation and Lymphangiogenesis

The lymphatic vessels serve as a conduit for transport of leukocytes and antigen-presenting cells to lymph nodes (LNs), which orchestrate initiation of adaptive immune response (11). LECs express the chemokine ligand, CCL21 that attracts and guides the interactions of CCR7-positive T, B, and dendritic cells (DCs) to LNs *via* the afferent lymphatics (53). Not all LECs are equal, reportedly, LN–LECs express different levels of CCL21 forming chemokine gradients that facilitate directional migration into the LNs through an atypical chemokine receptor, CCRL1 (54). The role of LECs in immune regulation has been demonstrated in a series of papers showing LECs contributing to the induction of peripheral tolerance of DC and T cells. In human LECs, tumor necrosis factor alpha (TNFα) induces vascular and intercellular cell adhesion molecule 1 (VCAM-1, ICAM-1) and E-selectin, facilitating adherence of DCs to the endothelium (55). TNFα-stimulated lymphatic endothelium can interact with DCs *via* cell-to-cell contact to suppress human DC maturation and function by an ICAM-1–Mac-1 (CD11b) interaction (56). Notably, murine LECs lack expression of co-stimulatory ligands but can express the inhibitory checkpoint ligand, programed cell death ligand-1 (PD-L1) to negatively regulate CD8^+^ T cells (57–60). LECs can also express MHC II *in vivo* and may induce tolerance of CD4^+^ and CD8^+^ T cells either by acting as an antigen reservoir for DCs or through cross-presentation of antigens (60–64). The mechanism of antigen transfer from LEC to DCs and whether LECs can induce similar levels of tolerance as DCs remains to be further understood.

During inflammation, the lymphatic system becomes activated and lymphatic remodeling is induced in both peripheral tissues and the draining LN (65). The increase in lymphangiogenesis may aid in the resolution of inflammation. Inflammation-induced lymphangiogenesis is commonly regulated by pathways involving VEGF-C/VEGFR-3 and VEGF-A/VEGFR-2 signaling (11). Studies in mice demonstrated that lymphangiogenesis is driven by increased VEGF-C, VEGF-D, and VEGF-A from macrophages during acute skin inflammation and chronic airway infection, reported to promote antigen clearance and prevent lymphedema (66, 67). Lymphatic vessels are impaired during chronic skin inflammation, which can be alleviated by the overexpression of VEGF-C (68). Interestingly, VEGF-C stimulation in skin inflammation instigated LECs to produce anti-inflammatory prostaglandin synthase, which led to higher levels of IL-10 on DCs leading to suppressed DC maturation (69). B cells can enhance the growth of LN lymphatic vasculature through VEGF secretion and increase DC migration to the LN (70). However, interferon-gamma (IFN-γ) secretion from T cells suppressed growth of LN-lymphatic vasculature *in vivo* and downregulated the expression of PROX1, LYVE-1, and podoplanin *in vitro* in a JAK/STAT-dependent mechanism (71). IFN-γ knockout mice express a higher baseline of lymphatic vasculature in the LN. Expression of PROX1, VEGFR-3, and LYVE-1 are also downregulated during acute skin inflammation (72, 73). In human dermal LECs, transforming growth factor-β (TGF-β) or TNFα stimulation results in loss of PROX1 and LYVE-1 expression (74, 75). In contrast, studies in mice suggest that NF-κB induces PROX1 and VEGFR-3 in a lipopolysaccharide (LPS)-induced peritonitis model, increasing sensitivity of pre-existing lymphatic vessels to VEGF-C and VEGF-D-expressing leukocytes (76). Additionally, IL-3 in LECs can induce PROX1 and podoplanin expression and maintain the differentiated LEC phenotype *in vitro* (77). LECs are also a major source of IL-7 *in vivo* which is required for remodeling and homeostasis of the LN microenvironment (78).

## miRNAs in LECs During Inflammation and Infection

Studies have demonstrated miRNAs in the regulation of inflammation including miR-146a/b, miR-155, and miR-132 in both immune and non-immune cell types (79–82). Several activities have been reported for miR-155 across the immune system, including Th1 differentiation of murine CD4^+^ T cells by inhibiting IFN-γ signaling (83) and production of immunoglobulin class-switch differentiation of B cells by targeting transcription factor PU.1 (84). A wide range of inflammatory stimuli induce miR-155 expression including LPS, poly (I:C), IFN-β, and TNFα in human and murine macrophages, monocytes, and endothelial cells (79, 80, 85, 86). In addition, miR-155 regulates angiogenesis and inflammation by negatively regulating ETS-1, upstream of VCAM-1, and angiotensin II type 1 receptor (87).

microRNA profiling of rat mesenteric LECs treated with TNFα for 2, 24, and 96 h indicated a distinct miRNA signature at various time points (88). Several miRNAs involved in angiogenesis, endothelial sprouting, and cell migration were upregulated, while miRNAs associated with cell survival and proliferation were downregulated at 24 and/or 96 h. Of those upregulated, miR-9 was shown to directly target NF-κB, downstream of TNFα signaling, and regulate TNFα-mediated inflammatory mechanisms. In addition, overexpression of miR-9 increases VEGFR-3 expression and tube formation, indicating a possible role in lymphangiogenesis. VEGFR-3 was also shown to be regulated by a mirtron miR-1236, arising from a spliced-out intron that is processed independently of Drosha, in human LECs (89). IL-1β can induce miR-1236 and downregulate VEGFR-3 protein which is similarly reported in inflammatory lymphangiogenesis. Although miR-1236 is lowly expressed in human LECs, it may be upregulated during inflammation-induced lymphangiogenesis to control the expression of VEGF-C/VEGFR-3 signaling.

## Lessons from Kaposi’s Sarcoma Herpesvirus (KSHV)

Our understanding of gene regulation in LECs has advanced significantly by studying infectious diseases that directly involve LECs. Kaposi’s sarcoma (KS) is a tumor from lymphatic endothelial origin and is the most common cancer in untreated HIV-positive patients (90). KSHV infects both LECs and BECs to induce transcriptional reprograming giving rise to mixed phenotypes of LECs and BECs (91, 92). Phenotypically, KS is most similar to LECs and occurs at sites rich in LECs such as skin, LN, and mucosa (92). KSHV infection of human LECs induces an early antiviral miRNA response from miR-132 and miR-146a and inhibition of these miRNAs suppressed viral gene expression (82). Overexpression of miR-132 negatively regulates inflammation by impairing the expression of IFN-β and interferon-stimulated gene 15. Upon KSHV infection, miR-132 targets the transcriptional co-activator EP300 and downregulates the interferon response, increasing viral gene expression. In addition, KSHV can influence endothelial cell motility by downregulating the miR-221/miR-222 cluster and upregulating miR-31 (93). Whether upregulation of miR-31 can regulate PROX1 during KSHV infection is unknown. A KSHV latent gene, kaposin B was found to stabilize PROX1 mRNA and drive lymphatic reprograming of BECs (50). An additional target of KSHV infection is the transcription factor c-MAF, which represses BEC-specific identity in human LECs (31, 91). Downregulation of MAF occurs early and is maintained throughout viral infection. The miR-155 KSHV ortholog, miR-K12-11 (94), was shown to regulate MAF in human LECs (31). Interestingly miR-155 has been shown to suppress MAF expression in murine CD4^+^ T cells (95).

## Concluding Remarks and Future Directions

**Figure 1:**
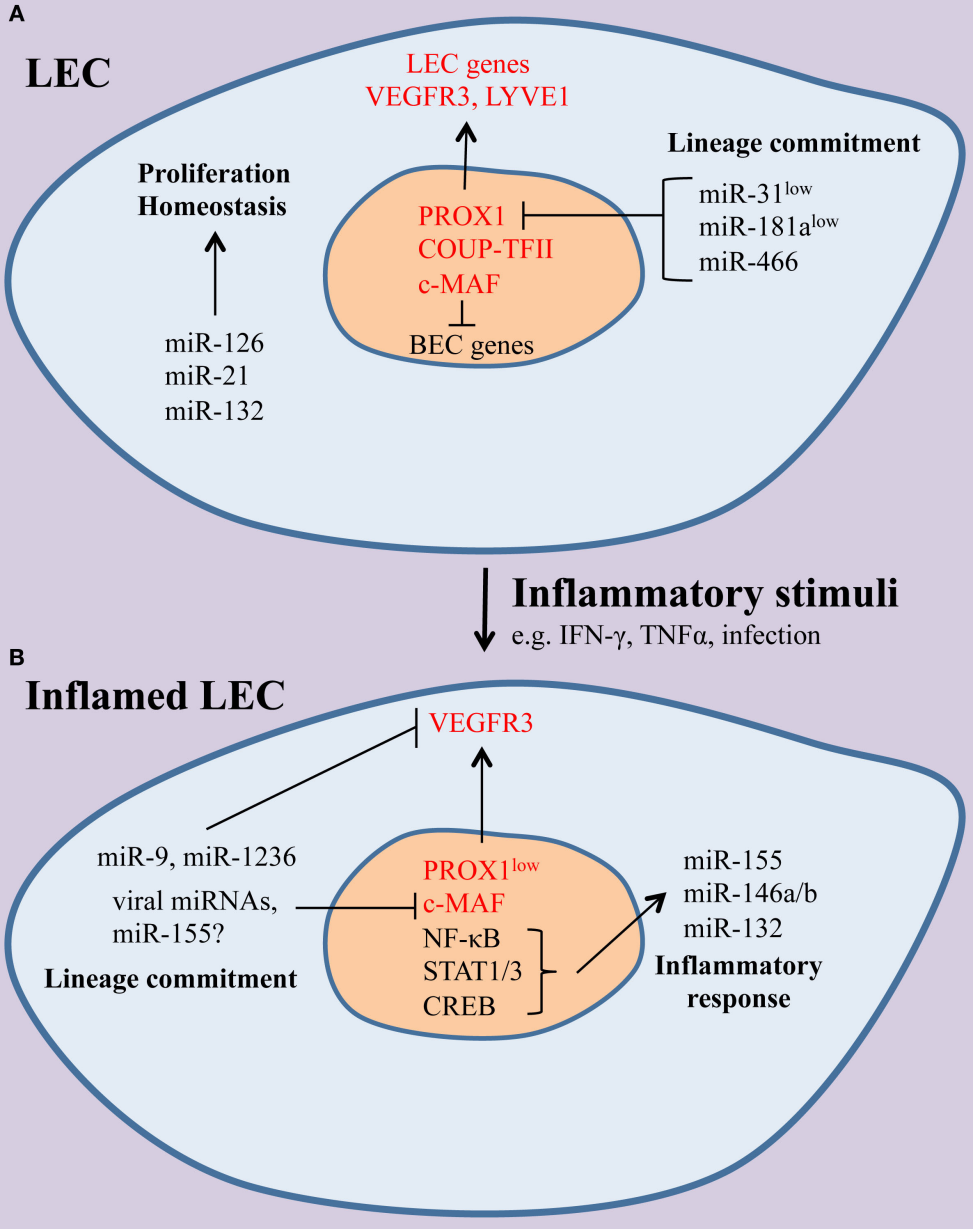
**The effect of inflammation on the microRNAs (miRNA) landscape of lymphatic endothelial cells (LECs)**. **(A)** Under homeostatic conditions, miRNAs, including miR-126, miR-21, and miR-132, contribute to normal LEC function. Lymphatic identity is maintained through suppression of the blood endothelial cell (BEC)-enriched miRNAs miR-31 and miR-181a, which can repress LEC-specific genes, including the master LEC fate regulator PROX1 and the receptor tyrosine kinase vascular endothelial growth factor receptor-3 (VEGFR-3). **(B)** During inflammation, a set of immunologically active miRNAs (miR-155, miR-132, miR-146a) are induced and shape LEC immune responses. In addition, LEC-specific genes are downregulated and miRNAs, including miR-9, miR-1236, and miR-K12-11, a viral ortholog of miR-155, contribute to the loss of LEC identity. It is likely that other miRNAs may modulate immune gene expression and lineage plasticity in LECs.

Our understanding of miRNAs in LEC activation has greatly increased from recent reports but this area remains understudied (Figure 1; Table 1). LEC plasticity is under miRNA regulation that allows the rapid response of lymphatic endothelium to inflammatory and angiogenic stimuli. LECs display heterogeneity, and there are different types of lymphatic vessels and LECs that have organ-specific functions (96). Studying miRNAs in certain types of lymphatic vessels and niches, such as the skin, LN, or subpopulations within these contexts, can introduce new tools to understand the different functions that LECs regulate in these tissues.

**Table 1:** **microRNAs (miRNAs) in the lymphatic endothelium**.

miRNA	Primary role	Function and target	Model system	Reference
miR-126	Angiogenesis	Highest expressed miRNA in endothelial cells, which regulates angiogenesis through SPRED1 and VCAM-1	Human primary ECs, murine ECs	Wang et al. (38), Harris et al. (37), and Fish et al. (39)
	Inflammation			
miR-132	Angiogenesis	Acts as an angiogenic switch by targeting p120RasGAP	Human umbilical vein ECs	Anand et al. (41)
	Inflammation	Regulates anti-viral immunity through EP300	Kaposi’s sarcoma herpesvirus (KSHV)-infected lymphatic endothelial cell (LECs)	Lagos et al. (82)
miR-9	Inflammation	Regulates vascular endothelial growth factor receptor-3 (VEGFR-3), lymphangiogenesis, and NF-κB signaling	Rat LECs and human primary LECs	Chakraborty et al. (88)
miR-1236	Inflammation	Induced by IL-1β and regulates VEGFR-3 and lymphangiogenesis	Cultured human dermal LECs	Jones et al. (89)
miR-181a	Lineage commitment	Blood endothelial cell (BEC)-expressed miRNA, which inhibits PROX1 in LEC development	Murine LECs	Kazenwadel et al. (22)
miR-31	Lineage commitment	BEC-expressed miRNA which inhibits PROX1 and FOXC2 in LEC development	Human primary LECs, xenopus, and zebrafish	Pedrioli et al. (40)
miR-466	Lineage commitment	Inhibits PROX1 and tube formation	HDLECs and corneal lymphatic vessels	Seo et al. (52)
miR-K12-6, miR-K12-11 (ortholog of miR-155)	Lineage commitment	Viral miRNAs that target c-MAF contributing to virus-induced LEC reprograming	KSHV-infected LECs	Hansen et al. (31) and Hong et al. (91)
miR-146a/b	Inflammation	Early-response miRNA involved in TLR4 signaling and innate immunity	KSHV-infected LECs	Lagos et al. (82)
miR-155	Inflammation	Targets ETS-1 upstream of endothelial adhesion molecules such as VCAM-1	Human umbilical vein ECs	Zhu et al. (87)
	Angiogenesis			
miR-221/miR-222	Angiogenesis	Targets transcription factors ETS-2 and ETS-1, respectively, regulating EC motility	Human primary LECs, KSHV-infected LECs	Wu et al. (93)

Targeting miRNAs such as miR-126, miR-9, and miR-132 (Table 1) presents a novel opportunity to deliver localized therapy for treating disease. This can be either to inhibit or mimic the function of the miRNA. Anti-miR-132 was shown to inhibit angiogenesis and decrease tumor burden in a mouse model of human breast carcinoma (41). Antagonism of miR-122 to treat hepatitis C virus infection is the first miRNA-targeting therapy in Phase II clinical trials (97). A challenge for miRNA-based therapies is ensuring effective delivery. Targeting miRNAs that drain into the LN through lymphatics vessels can lower the chances of off-target effects, drug resistance, and toxicity (98, 99). Lymphatic flow is unidirectional and the vessels can act as a bypass for absorption of compounds, such as lipophilic small molecule drugs, to avoid hepatic first-pass metabolism and enhance bioavailability (100). There are several routes that can be exploited for therapeutic delivery, including mucosal, intestinal, and parenteral (101). The lymphatic system is also thought to link the brain and the immune system (102). Although, lymphatic drug delivery is in its infancy, this approach may serve as a platform for accurately delivering miRNA-modifying compounds to target sites, providing new therapeutic opportunities for chronic inflammatory diseases.

## Author Contributions

DL, MC, DY conceived, co-wrote, and edited the mini review.

## Conflict of Interest Statement

The authors declare that the research was conducted in the absence of any commercial or financial relationships that could be construed as a potential conflict of interest.
